# Association between tooth loss and geriatric syndromes in older adults: a cohort study from a rural area in eastern China

**DOI:** 10.1007/s40520-025-03032-5

**Published:** 2025-04-18

**Authors:** Caihong He, Yihan Wang, Chengfan Qin, Nan Hua, Yichen Yang, Jing Chen, Qin Zhang

**Affiliations:** 1 Department of Geriatrics, The First Affiliated Hospital, School of Medicine, Zhejiang University, Hangzhou, China; 2 Key Laboratory of Diagnosis and Treatment of Aging and Physic-Chemical Injury Diseases of Zhejiang Province, The First Affiliated Hospital, School of Medicine, Zhejiang University, Hangzhou, Zhejiang 310003 China; 3 The First Affiliated Hospital of Zhejiang University School of Medicine, 79 Qingchun Road, Hangzhou City, Zhejiang Province 310003 China

**Keywords:** Tooth loss, Sarcopenia, Frailty, Malnutrition risk, Falls, Geriatric syndromes

## Abstract

**Background:**

Tooth loss was linked to health status, with substantial implications for malnutrition and chronic inflammation risks in older adults, especially among vulnerable groups. This study aimed to explore the associations between tooth loss severity, denture status, and geriatric syndromes.

**Methods:**

In 2019, 1094 participants were recruited and subjected to face-to-face interview to assess tooth loss severity, along with grip strength and body composition. In 2023, a follow-up was conducted with a subsample of the participants. Logistic regression analysis was utilized to explore the association between tooth loss severity at baseline and geriatric syndromes (sarcopenia, malnutrition risk, frailty, fall) at fourth year follow-up, as well as association between denture status and geriatric syndromes.

**Results:**

The multivariate analyses showed that having tooth loss affecting daily life at baseline was associated with a 1.80-fold higher prevalence of sarcopenia and 2.31-fold higher prevalence of malnutrition risk after four years. Participants with fewer than 10 teeth had significantly higher odds of geriatric syndromes compared to those with 21 or more teeth: 1.87-fold for sarcopenia (95% CI: 1.07 to 3.26), 2.99-fold for malnutrition risk (95% CI: 1.93 to 4.62), and 1.68-fold for frailty (95% CI: 1.10 to 2.56). Older adults with tooth loss who did not have dentures exhibited a significantly higher odds of sarcopenia, malnutrition risk, frailty, and falls, more number of geriatric syndromes.

**Conclusion:**

Higher severity level of tooth loss at baseline were associated with higher odds of geriatric syndromes at fourth year in older adults. Dentures partially mitigate the association between tooth loss and the higher odds of geriatric syndromes. Screening and intervening oral health is important for the prevention of geriatric syndromes in older adults.

**Clinical trial number:**

Not applicable.

**Supplementary Information:**

The online version contains supplementary material available at 10.1007/s40520-025-03032-5.

## Introduction

As the global population continues to age, the prevalence of individuals contending with multiple chronic conditions and frailty is on the rise, imposing a significant burden on society [1]. Oral health, an essential component of an individual’s overall well-being, is even more significant in the older adults [2]. Longitudinal studies from the United Kingdom and the United States have revealed a compelling link between oral health issues, particularly tooth loss and dry mouth, and the progression to frailty in later life in older adults [3].

Tooth loss is a common oral health problem among older adults, and its prevalence increases with age [4]. Alarmingly, in 2010, an estimated 158 million individuals, representing 2.3% of the global population, were devoid of natural teeth [5]. A closer examination within the United States from 2011 to 2016 revealed that among adults aged 50 and older who underwent dental examinations, 10.8% were edentulous, while 16.9% exhibited severe tooth loss [6]. The situation of missing teeth in the global population is very serious. The incidence of tooth loss was notably higher among demographic groups characterized by low socioeconomic status, limited education, disabilities, certain chronic diseases, living in solitude, or residing in rural settings [6–8]. Tooth loss, often the culmination of various oral pathologies such as advanced dental caries and severe periodontal disease [9], can be exacerbated by systemic conditions including diabetes mellitus and malnutrition [10–12]. Beyond its impact on aesthetics, tooth loss can trigger a cascade of physical and psychosocial issues [13].

Malnutrition, sarcopenia, frailty, and falls are common geriatric syndromes that frequently co-occur in the older adults [14]. Presence of these conditions would increase the risk of disability, hospitalization, mortality [15]. Nutritional deficiencies during the aging process are identified as pivotal risk factors for these adverse geriatric syndromes [16]. The loss of teeth compromises masticatory function, propelling older adults to favor soft foods and eschew fresh vegetables and fruits, thereby heightening the risk of nutritional deficiencies [4, 17]. Prior researches have established associations between tooth loss and a spectrum of conditions including sarcopenia, frailty, cardiovascular disease, cognitive impairment, social engagement, and Alzheimer’s disease [18–24]. However, these studies have predominantly employed cross-sectional methodologies or have examined the relationship between tooth loss and a single disease, with the majority of the study populations being urban dwellers or residents of nursing facilities [20, 23, 25–28]. As a result, research exploring the relationship between tooth loss and geriatric syndromes in the rural older adults remains scarce.

This study, conducted in a rural area from eastern China from 2019 to 2023, is a meticulously designed cohort study which focus on more geriatric syndromes. Our objective was to delineate the relationship between tooth loss and malnutrition risk, sarcopenia, frailty, and falls, which are pivotal geriatric syndromes impacting the older adults. Furthermore, the study was designed to explore the potential mitigating effects of denture use on these geriatric syndromes, an aspect that has been under-investigated in the existing literature. This endeavor is not only scientifically significant but also has profound implications for public health, particularly in the context of vulnerable rural older adults. By providing a new scientific basis for understanding the multifaceted implications of dental health on overall well-being, our study aims to contribute to the evidence base that can inform strategies for improving health outcomes in this often-overlooked segment of society.

## Methods

### Study design and participants

This was a well-designed cohort study, and participants for this present study were recruited from ten villages in Doumen, a township located in Shaoxing, Zhejiang Province, China, in the year 2019. The inclusion criteria for the cohort at baseline in 2019 were as follows: (1) older participants aged 60 years and over; (2) individuals who were long-term residents or workers in Doumen; (3) those with a life expectancy exceeding one year. The exclusion criteria included: (1) individuals with acute infectious diseases; (2) those in the acute phase of certain illnesses, such as myocardial infarction, cerebral infarction, or acute exacerbation of chronic obstructive pulmonary disease; (3) individuals with significant psychiatric problems or severe cognitive decline who are unable to cooperate with questionnaires and somatic function assessments.

In 2019, a total of 1386 participants from ten villages in Doumen were recruited for the cohort at baseline. Participants with incomplete data regarding body composition, handgrip strength, or tooth loss were excluded and 1094 participants at baseline (79.93%) were included to examine the association between severity of tooth loss and geriatric syndromes using baseline data (study sample 1). In 2023, a sub-sample of participants from five of the original villages were followed up, and 685 participants (61.75%) participated in the follow-up assessment. The 685 participants (study sample 2) were included in the analysis to assess the association between number of teeth, denture status and geriatric syndromes using cross-sectional data collected in 2023. In total, 526 (study sample 3) out of the 685 participants had completed data on baseline tooth loss assessment and health outcomes at follow-up, who were included for the analysis to assess the association between baseline tooth loss severity and the presence of geriatric syndromes at the fourth year follow-up. We show this process in Figure S1 of the supplementary materials.

Both in 2019 and 2023, data collection procedures included questionnaire interviews, handgrip strength measurements, and body composition assessments. These questionnaires were developed for this study and the original questionnaires have been provided in the supplementary materials. Additionally, during the 2023 follow-up, assessments for frailty, malnutrition risk, and the incidence of falls were conducted, complemented by dental examinations.

This study was approved by the Ethics Committee of the First Affiliated Hospital of Zhejiang University Medical College (Reference Number: 20191276). All participants were fully informed about the study protocol and provided written informed consent before participating in the study. Participants who were illiterate had their fingerprints taken under the supervision of Doumen Community Health Center staff or family members.

## Measurments

### Measurments of handgrip strength, muscle mass and sarcopenia

Handgrip strength was evaluated using the Jamar Hydraulic Hand Dynamometer (model 5030J1, Sammons Preston, Inc., Bolingbrook, Illinois, USA), with participants’ elbows positioned at a 90-degree angle of flexion. Each participant’s grip strength was measured twice for both the left and right hands, and the highest value from these measurements was selected for subsequent analysis [16]. Grip strength below 28 kg for men and below 18 kg for women was considered low, in accordance with the Asian Working Group for Sarcopenia (AWGS) criteria from 2019 [29].

Body composition was assessed using the Tsinghua Tong fang Body Composition Analyzer (Tsinghua Tong fang Corporation, Beijing, China), which features an 8-point tactile electrode-impedance meter. Participants were directed to stand upright on the electrodes, grasp the analyzer’s handles, and slightly abduct their arms while minimizing movement during the measurement process [30]. The sum of the appendicular skeletal muscle mass (ASM) in kilograms, as indicated on the assessment report, was utilized as a direct measure of ASM. The height-adjusted appendicular skeletal muscle mass index (ASMI, calculated as ASM divided by the square of height in meters, ASMI = ASM/height^2^) was employed to evaluate muscle mass. A low ASMI, defined as less than 7 kg/m^2^ for men and less than 5.7 kg/m^2^ for women, was used to identify reduced muscle mass, following the AWGS 2019 guidelines [16]. The diagnosis of sarcopenia within this study adhered to the AWGS 2019 Revised Consensus and Critical Values, requiring the concurrent presence of both a low appendicular skeletal muscle mass index (ASMI) and reduced handgrip strength (HG) to establish a diagnosis of sarcopenia [16].

### Definitions of malnutrition risk, frailty, fall and total outcome score

The Mini Nutritional Assessment-Short Form (MNA-SF) [31] was employed as a screening instrument for the risk of malnutrition, with a cutoff score of ≤ 11 indicating the presence of malnutrition risk, and scores above this threshold suggesting adequate nutritional status. The FRAIL scale, a validated tool for assessing frailty in community-dwelling older adults, was utilized to determine the frailty status of participants [32]. The FRAIL scale is composed of five items: fatigue, resistance, ambulation, illness, and loss of weight. Five questions on the scale were answered “yes” (1 point) or “no” (0 points). The total score is five points. A score of ≥ 3 on the FRAIL scale was considered indicative of frailty [33]. Participants were queried about their fall history over the past year, specifically asking if they had experienced any type of fall, including but not limited to falling, wrestling, slipping, tripping, or falling from a horizontal surface. A fall was operationally defined as a sudden, involuntary, unintentional change in body position resulting in contact with the ground. Participants who reported one or more falls were noted as having experienced falls in the past year.

To encapsulate the overall health status of the older participants, a scoring system was developed based on the four key indicators: sarcopenia, frailty, malnutrition risk, and falls. Each indicator was assigned a score of 0 or 1, with 1 indicating the presence of the condition and 0 indicating its absence. The scores for these four indicators were then aggregated to form the “Total Outcome Score”, which ranged from 0 to 4. Higher scores on “Total Outcome Score” indicate a higher number of comorbid geriatric syndromes and a poorer, more distressing physical state for the older people [34–36].

### Tooth loss

Tooth loss was evaluated at both baseline in 2019 and follow-up in 2023. At baseline (2019), participants had a face-to-face interview with a trained medical staff to assess the severity of tooth loss, and would be asked: “Do you have any missing teeth?” Participants were then required to select one of the following options: (1) No; (2) Yes, but it does not affecting daily life; (3) Yes, and it significantly affecting my daily life. This type of questioning had also been used in previous studies to assess the severity of tooth loss [24, 37, 38]. At the 2023 follow-up, oral health was assessed by trained medical staff to evaluate the number of teeth remaining and the status of dentures among participants. Any retained roots were excluded from the count of remaining teeth. Participants were stratified into six groups based on their remaining teeth: (1) less than five teeth; (2) five to ten teeth; (3) 11 to 15 teeth; (4) 16 to 20 teeth; (5) 21 to 25 teeth; (6) more than 25 teeth.

Additionally, participants were asked and examined about the presence of any dental prosthetics, with options including: (1) Without dentures; (2) Dental implants or fixed dentures; (3) Removable partial dentures; (4) Complete removable dentures [39]. For the purpose of regression analysis, the latter three categories were consolidated into a single group, denoting the presence of any form of dentures.

### Demographic and health information

Demographic information, lifestyle habits, and the prevalence of chronic diseases were meticulously recorded at both baseline and follow-up assessments. Age was stratified into three distinct groups for analytical purposes: 60–70 years, 70–80 years, and greater than 80 years. Participants reported their smoking and drinking status, which were categorized as “Never”, “Former”, or “Current” to reflect their habits. Because of the generally low level of education of the participants, educational level was classified into “Illiterate”, “Primary” or “Secondary and above” to capture the spectrum of educational backgrounds. We inquired about the presence of a comprehensive list of chronic conditions, including but not limited to hypertension, diabetes mellitus, heart disease, stroke or cerebrovascular disease, Parkinson’s disease, dementia, chronic obstructive pulmonary disease (COPD), tuberculosis, cancer, gastrointestinal disease, metabolic disorders, chronic nephritis, cataracts, and history of falls. Based on the characteristics of the distribution of these chronic diseases in the study population, the number of chronic diseases was categorized into “No disease”, “One chronic disease”, “Two chronic diseases” and “More than two chronic diseases” [40].

## Statistical methods

In participant characterization, categorical variables were reported as frequencies and percentages, and continuous variables following a normal distribution were expressed as mean ± standard deviation. Comparisons between groups for categorical variables were made using the chi-square (χ²) test, while comparisons between two groups for continuous variables were made using the independent samples t-test. We employed logistic regression analysis to investigate the relationship between the severity of tooth loss at baseline and various geriatric syndromes at the fourth-year follow-up, including sarcopenia, frailty, malnutrition risk, falls, and the total outcome score. Tooth loss severity was dichotomized into two categories for analysis: “Without tooth loss or tooth loss not affecting life” and “Tooth loss affecting life”. The former category was utilized as the reference group in the analysis. The analysis accounted for possible confounding factors, including age group (60–70 years, 70–80 years, > 80 years), sex, education level (illiterate, primary, secondary school or above), drinking status, smoking status, and the number of chronic diseases. Selection of covariates adjusted in the models was based on previous literature or statistical significance of an association of the selected variable with both the outcome and the exposure of interest [6, 22, 24], we adjusted the variables age group and sex in Model 1; age group, sex and education level in Model 2; and age group, sex, education level, smoking status, drinking status and the number of chronic diseases in Model 3. To assess the goodness of fit of the logistic regression model, we used the Hosmer-Lemeshow test. The p-value of the test result was greater than 0.05, indicating that there was no significant difference between the predicted values of the model and the actual observed values, suggesting that the model was well-fitted. To ensure the robustness of our findings, a cross-sectional analysis was conducted using data solely from the 2023 wave. This analysis examined the association between the number of teeth and the same set of geriatric syndromes. The number of teeth was categorized into three groups for this analysis: 21 or more teeth, 11–20 teeth, and 10 or fewer teeth [23]. Additionally, logistic regression analysis was conducted to explore the association between the use of dentures and geriatric syndromes using 2023 data. Participants were categorized into five groups based on number of tooth and denture status: (1) teeth ≥ 21; (2) teeth 11–20 with dentures; (3) teeth 11–20 without dentures; (4) teeth ≤ 10 with dentures; (5) teeth ≤ 10 without dentures. A p-value of less than 0.05 was considered to indicate statistical significance. All statistical tests were conducted using STATA version 18.

## Results

### Participants characteristics

The characteristics of the 1,094 participants and the severity of their tooth loss are presented in Table 1. Compared to those without tooth loss, those having tooth loss affecting daily life were older, and had a higher percentage of sarcopenia (8.66% vs. 20.60%).

**Table 1:** Characteristics of 1094 participants enrolled at baseline in 2019

Characteristic	No tooth loss (*n* = 127)	Having tooth loss that not affected life (*n* = 734)	Having tooth loss that affected life (*n* = 233)	*p*
Sex, %				0.25
Female	47.79	47.73	53.95	
Male	52.21	52.27	46.05	
Age group (year), %				< 0.001**
60–70	60.63	47.95	26.18	
70–80	34.65	42.51	47.64	
> 80	4.72	9.54	26.18	
Education level, %				0.18
Illiterate	48.82	50.90	57.94	
Primary	40.94	40.14	36.91	
Secondary school or above	10.24	8.96	5.15	
Drinking, %				0.04*
Never	51.18	58.12	62.93	
Current	45.67	34.65	30.60	
Ever	3.15	7.23	6.47	
Smoking, %				0.58
Never	70.86	73.50	74.14	
Current	20.48	18.03	14.66	
Ever	8.66	8.47	11.20	
Sarcopenia, %				< 0.001**
No	91.34	87.74	79.40	
Yes	8.66	12.26	20.60	
Number of chronic disease, %				0.59
None	22.12	18.89	18.86	
One	34.52	40.34	37.72	
Two	30.97	24.43	25.44	
More than two	12.39	16.34	17.98	

Notes: Data presented are mean ± SD, median (IQR), or N (%). SD: standard deviation. Data in bold indicate statistically significant values; **p < 0.001, *p < 0.05

Of the initial 1,094 participants, 529 were followed up at the fourth year. Of these, the highest percentage was frailty (35.73%), followed by the malnutrition risk (33.84%). The percentages of sarcopenia and a history of falls within the past year were relatively lower, (14.37% vs. 17.39%). Detailed data are available in Supplementary Table S1. Supplementary Table S2 provides a profile of the 685 participants who were followed up in 2023, the percentages of sarcopenia, frailty, malnutrition risk, and falls were similar to those presented in Supplementary Table S1. Table 2 illustrates a higher prevalence of individuals with fewer than 10 teeth among the oldest age group (80 + years), females, and those with illiteracy. Additionally, a higher percentage of denture use was observed among individuals with fewer than 10 teeth.

**Table 2:** Tooth number and denture status assessed at follow-up in 2023 in the study sample

Characteristic	Teeth ≥ 21 (*n* = 231)	Teeth 11–20 (*n* = 165)	Teeth ≤ 10 (*n* = 289)
Sex, %			
Female	49.35	49.70	63.32
Male	50.65	50.30	36.68
Age group (year), %			
60–70	42.42	28.48	19.38
70–80	51.51	56.36	50.52
> 80	6.07	15.16	30.10
Education level, %			
Illiterate	46.29	45.96	62.15
Primary	41.05	42.24	31.25
Secondary school or above	12.66	11.80	6.60
Having dentures, %			
Having dentures	35.06	51.52	68.86
No dentures	64.94	48.48	31.14

### Associations between tooth loss severity and geriatric syndromes

The regression analysis showed that higher severity level of tooth loss at baseline were associated with higher odds of geriatric syndromes at the fourth year, including sarcopenia, malnutrition risk, and total outcome score (Table 3). Compared to those without tooth loss or with tooth loss not affecting daily life, participants with tooth loss affecting daily life at baseline demonstrated a significant higher prevalence of geriatric syndromes at the fourth year follow up. After adjusting for age, sex, education, drinking, smoking, number of chronic disease, severe tooth loss that affecting life was associated with a 1.80-fold higher odds of sarcopenia (95% CI: 1.02 to 3.21), a 2.31-fold higher odds of malnutrition risk (95% CI: 1.44 to 3.69), and as well as higher total outcome score (OR 2.25, 95% CI: 1.33 to 3.82). However, the severity of tooth loss was not found to be associated with frailty or falls in the past year. Using 2019 data only to assess the relationship between the severity of tooth loss and sarcopenia showed consistent results (Supplementary Table S3). The adjusted OR for sarcopenia in participants with tooth loss affecting daily life was 2.91 (95% CI: 1.42 to 5.97).

**Table 3:** Association between severity of tooth loss in 2019 and geriatric syndromes at the 4th-year follow-up in 2023 among 529 participants

Variables	Non-adjusted OR (95% CI)	Model 1 OR (95% CI)	Model 2 OR (95% CI)	Model 3 OR (95% CI)
Severity of tooth loss and sarcopenia				
No tooth loss or having tooth loss that not affecting life (*N* = 429)	1 (reference)	1 (reference)	1 (reference)	1 (reference)
Having tooth loss that affecting life (*N* = 100)	**2.29 (1.33**, 3.94)*	**1.90 (1.09**, 3.33)*	**1.90 (1.08**, 3.33)*	**1.80 (1.02**, 3.21)*
Severity of tooth loss and malnutrition risk				
No tooth loss or having tooth loss that not affecting life (*N* = 429)	1 (reference)	1 (reference)	1 (reference)	1 (reference)
Having tooth loss that affecting life (*N* = 100)	**2.41 (1.54**, 3.77)**	**2.39 (1.51**, 3.78)**	**2.40 (1.52**, 3.82)**	**2.31 (1.44**, 3.69)**
Severity of tooth loss and frailty				
No tooth loss or having tooth loss that not affecting life (*N* = 429)	1 (reference)	1 (reference)	1 (reference)	1 (reference)
Having tooth loss that affecting life (*N* = 100)	1.53 (0.97, 2.40)	1.30 (0.81, 2.08)	1.30 (0.81, 2.09)	1.37 (0.84, 2.23)
Severity of tooth loss and fall				
No tooth loss or having tooth loss that not affecting life (*N* = 429)	1 (reference)	1 (reference)	1 (reference)	1 (reference)
Having tooth loss that affecting life (*N* = 100)	1.27 (0.72, 2.22)	1.11 (0.62, 1.98)	1.12 (0.62, 2.00)	1.06 (0.58, 1.94)
Severity of tooth loss and total outcome score				
No tooth loss or having tooth loss that not affecting life (*N* = 429)	1 (reference)	1 (reference)	1 (reference)	1 (reference)
Having tooth loss that affecting life (*N* = 100)	**2.54 (1.53**, 4.23)**	**2.33 (1.39**, 3.91)*	**2.29 (1.36**, 3.86)*	**2.25 (1.33**, 3.82)*

Notes: Model 1 adjusted for age, sex; Model 2 adjusted for age, sex, education; Model 3 adjusted for age, sex, education, drinking, smoking, number of chronic disease

Data in bold indicate statistically significant values: *p < 0.05,**p < 0.001

Hosmer-Lemeshow goodness-of-fit: all *p* > 0.05. A p-value > 0.05 indicates that the model fits the data well

CI: confidence interval

### Associations between number of teeth and geriatric syndromes

Table 4 presents the regression analysis assessing the associations between the number of teeth and health outcomes using 2023 data. A significantly higher odds of sarcopenia, frailty, malnutrition risk, and falls was observed for individuals with fewer than 10 teeth. In univariate analysis, the odds ratios for these outcomes were 2.87 for sarcopenia (95% CI: 1.71 to 4.81), 2.08 for frailty (95% CI: 1.40 to 3.08), 2.80 for having malnutrition risk (95% CI: 1.87 to 4.18), and 1.76 for having fall history over the past year (95% CI: 1.10 to 2.82), respectively, compared to those with 21 or more teeth. After adjustment for age, sex, education, drinking, smoking, number of chronic disease, the associations between the number of teeth and sarcopenia, frailty, and malnutrition risk remained significant, while the association with falls became insignificant. The total outcome score was 3.02 times higher (95% CI: 2.14 to 4.26) in the group with ≤ 10 teeth compared to the group with ≥ 21 teeth, suggesting that people with fewer than 10 teeth had a higher number of comorbid geriatric syndromes and were in poorer health.

**Table 4:** Association between number of teeth and geriatric syndromes among 685 participants using cross-sectional data collected in 2023

Variables	Non-adjusted OR (95% CI)	Model 1 OR (95% CI)	Model 2 OR (95% CI)	Model 3 OR (95% CI)
Number of teeth and sarcopenia				
≥ 21 (*N* = 231)	1 (reference)	1 (reference)	1 (reference)	1 (reference)
11–20 (*N* = 165)	1.24 (0.65, 2.37)	1.02 (0.53, 1.98)	1.01 (0.52, 1.97)	1.04 (0.53, 2.02)
≤ 10 (*N* = 289)	**2.87 (1.71**, 4.81)*	**2.01 (1.17**, 3.47)*	**1.90 (1.09**, 3.29)*	**1.87 (1.07**, 3.26)*
Number of teeth and malnutrition risk				
≥ 21 (*N* = 231)	1 (reference)	1 (reference)	1 (reference)	1 (reference)
11–20 (*N* = 165)	1.32 (0.82, 2.14)	1.32 (0.82, 2.14)	1.40 (0.86, 2.28)	1.42 (0.87, 2.32)
≤ 10 (*N* = 289)	**2.80 (1.87**, 4.18)*	**2.72 (1.79**, 4.14)*	**2.80 (1.86**, 4.42)*	**2.99 (1.93**, 4.62)*
Number of teeth and frailty				
≥ 21 (*N* = 231)	1 (reference)	1 (reference)	1 (reference)	1 (reference)
11–20 (*N* = 165)	1.46 (0.92, 2.31)	1.32 (0.83, 2.10)	1.26 (0.79, 2.02)	1.32 (0.82, 2.13)
≤ 10 (*N* = 289)	**2.08 (1.40**, 3.08)*	**1.67 (1.11**, 2.52)*	**1.56 (1.03**, 2.37)*	**1.68 (1.10**, 2.56)*
Number of teeth and fall				
≥ 21 (*N* = 231)	1 (reference)	1 (reference)	1 (reference)	1 (reference)
11–20 (*N* = 165)	1.01 (0.56, 1.82)	0.97 (0.53, 1.75)	0.98 (0.53, 1.77)	0.97 (0.53, 1.76)
≤ 10 (*N* = 289)	**1.76 (1.10**, 2.82)*	**1.66 (1.01**, 2.72)*	1.57 (0.95, 2.59)	1.51 (0.91, 2.51)
Number of teeth and total outcome score				
≥ 21 (*N* = 231)	1 (reference)	1 (reference)	1 (reference)	1 (reference)
11–20 (*N* = 165)	1.40 (0.95, 2.07)	1.32 (0.89, 1.95)	1.35 (0.91, 2.01)	1.38 (0.92, 2.07)
≤ 10 (*N* = 289)	**3.02 (2.14**, 4.26)**	**2.49 (1.74**, 3.58)**	**2.45 (1.70**, 3.52)**	**2.65 (1.83**, 3.83)**

Notes: Model 1 adjusted for age, sex; Model 2 adjusted for age, sex, education; Model 3 adjusted for age, sex, education, drinking, smoking, number of chronic disease

Data in bold indicate statistically significant values: *p < 0.05,**p < 0.001.

Hosmer-Lemeshow goodness-of-fit: all *p* > 0.05. A p-value > 0.05 indicates that the model fits the data well

CI: confidence interval

### Associations between denture status and geriatric syndromes

Older people with tooth loss who did not have dentures exhibited a significantly higher odds of sarcopenia, malnutrition risk, frailty, falls, and more number of geriatric syndromes, particularly among those with fewer than 10 teeth (Table 5). After adjustment for age group, sex, education level, drinking, smoking, and the number of chronic diseases, compared to those with ≥ 21 teeth, older individuals with fewer than 10 teeth and without dentures had a 3.25-fold higher odds of sarcopenia (95% CI: 1.68 to 6.28), a 3.64-fold higher odds of malnutrition risk (95% CI: 2.06 to 6.44), a 2.41-fold higher odds of frailty (95% CI: 1.36 to 4.26), and a 2.01-fold higher odds of having fall history over the past year (95% CI: 1.06 to 3.83). Interestingly, dentures were found to reduce the odds of sarcopenia, malnutrition risk, falls, and frailty in older individuals with ≤ 10 teeth. However, compared to those with ≥ 21 teeth, individuals with fewer than 10 teeth and with dentures still had 1.36-fold higher odds of sarcopenia (95% CI: 0.74 to 2.48), 2.63-fold higher odds of malnutrition risk (95% CI: 1.66 to 4.16), 1.39-fold higher odds of falls (95% CI: 0.80 to 2.38), and 1.36-fold higher odds of frailty (95% CI: 0.86 to 2.13). The odds were significant higher for those with more teeth loss but without dentures. For example, the OR was 4.01 for total geriatric syndromes outcome score (95% CI: 2.17 to 7.42) for those with teeth ≤ 10 without dentures as compared to those who had ≥ 21 teeth.

**Table 5:** Association between denture status and geriatric syndromes among 685 participants using cross-sectional data collected in 2023

Variables	Non-adjusted OR (95% CI)	Model 1 OR (95% CI)	Model 2 OR (95% CI)	Model 3 OR (95% CI)
Dentures and sarcopenia				
Teeth ≥ 21 (*N* = 231)	1 (reference)	1 (reference)	1 (reference)	1 (reference)
Teeth 11–20 with Dentures (*N* = 85)	0.59 (0.22, 1.62)	0.52 (0.19, 1.44)	0.52 (0.19, 1.45)	0.52 (0.19, 1.43)
Teeth 11–20 without Dentures (*N* = 80)	2.02 (0.98, 4.16)	1.57 (0.75, 3.31)	1.64 (0.77, 3.46)	1.63 (0.77, 3.46)
Teeth ≤ 10 with Dentures (*N* = 199)	**2.03 (1.15**, 3.58)*	1.39 (0.76, 2.53)	1.36 (0.74, 2.48)	1.36 (0.74, 2.50)
Teeth ≤ 10 without Dentures (*N* = 90)	**5.24 (2.83**, 9.70)**	**3.44 (1.80**, 6.61)**	**3.23 (1.68**, 6.25)**	**3.25 (1.68**, 6.28)**
P value for trend	**< 0.001**	**< 0.001**	**0.001**	**0.001**
Dentures and malnutrition risk				
Teeth ≥ 21 (*N* = 231)	1 (reference)	1 (reference)	1 (reference)	1 (reference)
Teeth 11–20 with Dentures (*N* = 85)	1.18 (0.65, 2.15)	1.21 (0.66, 2.10)	1.26 (0.69, 2.31)	1.27 (0.69, 2.33)
Teeth 11–20 without Dentures (*N* = 80)	1.49 (0.28, 2.68)	1.47 (0.81, 2.68)	1.57 (0.86, 2.87)	1.61 (0.88, 2.96)
Teeth ≤ 10 with Dentures (*N* = 199)	**2.51 (1.62**, 3.88)**	**2.57 (1.63**, 4.06)**	**2.63 (1.66**, 4.16)**	**2.79 (1.75**, 4.46)**
Teeth ≤ 10 without Dentures(*N* = 90)	**3.51 (2.07**, 5.96)**	**3.49 (2.00**, 6.08)**	**3.54 (2.01**, 6.21)**	**3.64 (2.06**, 6.44)**
P value for trend	**< 0.001**	**< 0.001**	**< 0.001**	**< 0.001**
Dentures and fall				
Teeth ≥ 21 (*N* = 231)	1 (reference)	1 (reference)	1 (reference)	1 (reference)
Teeth 11–20 with Dentures (*N* = 85)	0.83 (0.38, 1.83)	0.78 (0.35, 1.74)	0.82 (0.37, 1.83)	0.79 (0.35, 1.77)
Teeth 11–20 without Dentures (*N* = 80)	1.20 (0.59, 2.41)	1.14 (0.56, 2.30)	1.13 (0.55, 2.29)	1.17 (0.57, 2.40)
Teeth ≤ 10 with Dentures (*N* = 199)	1.56 (0.93, 2.62)	1.39 (0.81, 2.39)	1.39 (0.80, 2.38)	1.33 (0.76, 2.31)
Teeth ≤ 10 without Dentures(*N* = 90)	**2.21 (1.21**, 4.06)*	**2.00 (1.06**, 3.79)*	**2.04 (1.07**, 3.86)*	**2.01 (1.06**, 3.83)*
P value for trend	**0.010**	**0.046**	**0.045**	0.062
Dentures and frailty				
Teeth ≥ 21 (*N* = 231)	1 (reference)	1 (reference)	1 (reference)	1 (reference)
Teeth 11–20 with Dentures (*N* = 85)	1.00 (0.55, 1.82)	0.94 (0.51, 1.71)	0.86 (0.46, 1.59)	0.97 (0.51, 1.83)
Teeth 11–20 without Dentures (*N* = 80)	**2.07 (1.19**, 3.59)*	**1.80 (1.02**, 3.16)*	**1.81 (1.02**, 3.20)*	**1.94 (1.07**, 3.52)*
Teeth ≤ 10 with Dentures (*N* = 199)	**1.76 (1.15**, 2.70)*	1.38 (0.88, 2.17)	1.36 (0.86, 2.13)	1.52 (0.95, 2.43)
Teeth ≤ 10 without Dentures(*N* = 90)	**2.95 (1.75**, 4.97)**	**2.24 (1.30**, 3.88)*	**2.19 (1.26**, 3.80)*	**2.41 (1.36**, 4.26)*
P value for trend	**< 0.001**	**0.005**	**0.006**	**0.002**
Dentures and total outcome score				
Teeth ≥ 21 (*N* = 231)	1 (reference)	1 (reference)	1 (reference)	1 (reference)
Teeth 11–20 with Dentures (*N* = 85)	1.11 (0.66, 1.86)	1.07 (0.64, 1.80)	1.09 (0.64, 1.84)	1.16 (0.68, 1.98)
Teeth 11–20 without Dentures (*N* = 80)	**1.88 (1.10**, 3.20)*	**1.76 (1.02**, 3.01)*	**1.84 (1.06**, 3.19)*	**1.89 (1.08**, 3.33)*
Teeth ≤ 10 with Dentures (*N* = 199)	**2.65 (1.75**, 4.00)**	**2.31 (1.50**, 3.55)**	**2.31 (1.50**, 3.57)**	**2.58 (1.65**, 4.02)**
Teeth ≤ 10 without Dentures(*N* = 90)	**4.57 (2.54**, 8.20)**	**3.90 (2.13**, 7.11)**	**3.78 (2.06**, 6.93)**	**4.01 (2.17**, 7.42)**
P value for trend	**< 0.001**	**< 0.001**	**< 0.001**	**< 0.001**

Notes: Model 1 adjusted for age, sex; Model 2 adjusted for age, sex, education; Model 3 adjusted for age, sex, education, drinking, smoking, number of chronic disease

Data in bold indicate statistically significant values: *p < 0.05,**p < 0.001

Hosmer-Lemeshow goodness-of-fit: all *p* > 0.05. A p-value > 0.05 indicates that the model fits the data well

CI: confidence interval

## Discussion

In the present study, we found that higher severity level of tooth loss at baseline were associated with higher odds of various geriatric syndromes at fourth year in older adults, including sarcopenia, malnutrition risk, and the total outcome score. Tooth loss is more severe among women and illiterate individuals. Notably, malnutrition risk and sarcopenia were markedly elevated in individuals experiencing tooth loss that significantly affecting daily life. Additionally, less number of teeth was found to be a critical factor, with those having fewer than 10 teeth exhibiting a significantly increased odds of sarcopenia, frailty, malnutrition risk, and falls. Dentures were associated with decreased odds of these geriatric syndromes, especially among older adults with fewer than 10 teeth.

In this cohort study, we observed a significant association between tooth loss and an increased odds of geriatric syndromes. Our cohort study used face-to-face interviews to assess the severity of tooth loss and to categorize it by its impact on daily life. This approach, while generalized, considers not only the reduced number of teeth affecting eating but also the broader implications for oral function. Missing teeth can affect individuals in multiple ways beyond eating, such as causing tooth pain, gum inflammation, or a diminished ability to bite [41].

There were significant dose-response between higher severity of tooth loss and higher number of geriatric syndromes. Among the associations examined between tooth loss and geriatric syndromes, the effect sizes were profound for the increased odds of malnutrition and sarcopenia. While sarcopenia, frailty, malnutrition, and falls are distinct conditions, they share overlapping and synergistic relationships and also share common pathogenic mechanisms over ageing process [42], they are classic geriatric syndromes. Sarcopenia and frailty, both characterized by a decrease in muscle mass and physical function [43], increase the risk of falls, with malnutrition being a significant contributing factor in the development of both syndromes [15, 44]. Similarly, a study of Kiuchi et al. [45] showed that tooth loss is associated with the onset of dementia, with malnutrition partially mediating this association. So do we guess that malnutrition mediates the relationship between tooth loss and sarcopenia and frailty? Future research is needed to clarify the underlying mechanisms and strengthen the evidence base regarding the associations between tooth health and the development of geriatric syndromes in older adults.

Our findings showed that dentures were associated with decreased odds of geriatric syndromes, especially when the number of teeth is less than 10, suggesting that wearing appropriate dentures is important for preventing geriatric syndromes among those had tooth loss. The use of dentures to restore masticatory function is one of the most effective interventions for improving nutritional intake in older individuals with substantial tooth loss [46]. Denture use has been found to be associated with higher protein intake in older adults with tooth loss [47]. A study utilizing the Chinese Longitudinal Healthy Longevity Survey data found that denture use mitigated the adverse effects of tooth loss on all-cause and cause-specific mortality [27], which is consistent with our results.

The potential of dentures to enhance geriatric syndromes for older individuals experiencing tooth loss is evident; however, they do not fully replicate the benefits of natural dentition. In our study it was found that having dentures with fewer than 10 teeth was still associated with an increased risk of geriatric syndromes. This suggests that tooth loss is associated with geriatric syndromes not only because of decreased chewing function and inadequate nutritional intake, but possibly other causes such as chronic inflammation and oral microbiome dysbiosis, which are common precipitating factors for sarcopenia, frailty, and falls [48–51]. Therefore, in conjunction with denture recommendations, the provision of oral health education is deemed equally crucial for older adults at higher risk of tooth loss. Our study population, located in rural China, typically exhibits lower levels of education and poor lifestyle habits, such as smoking, drinking, and irregular tooth brushing. These people tend to have worse oral health status, and developing and implementing feasible oral health education programs for these older adults is essential.

This study, while providing valuable insights, had several limitations. Firstly, the initial phase of the research did not include a comprehensive examination of the dental status, specifically the count of remaining teeth, nor did it assess the risks of malnutrition, frailty, and falls, which are critical factors in the health profile of the older people. Secondly, the study was subject to a higher-than-anticipated rate of participant attrition, which may have affected the robustness of the findings. A larger initial sample size and an extended follow-up period could have potentially enhanced the study’s statistical power and the longevity of its conclusions.

## Conclusion

Tooth loss is significantly more severe among women and illiterate individuals in the study sample from rural areas. Higher severity level of tooth loss at baseline were associated with higher odds of geriatric syndromes at fourth year in older adults. Thus, screening and intervening oral health is important for the prevention of geriatric syndromes. Dentures partially mitigate the association between tooth loss and the higher odds of geriatric syndromes, suggesting that wearing appropriate dentures is important for preventing geriatric syndromes among older adults who had tooth loss.

## Electronic supplementary material

Below is the link to the electronic supplementary material.

Supplementary Material 1

## Data Availability

The datasets analysed during the current study are not publicly available due unpublished but are available from the corresponding author on reasonable request.
