# Supplementary material for: Association between tooth loss and geriatric syndromes in older adults: a cohort study from a rural area in eastern China
Source: Aging Clin Exp Res. 2025 Apr 18;37(1):128. doi: 10.1007/s40520-025-03032-5 (PMC12008081; doi:10.1007/s40520-025-03032-5)

**Figue S1. Flowchart**

**
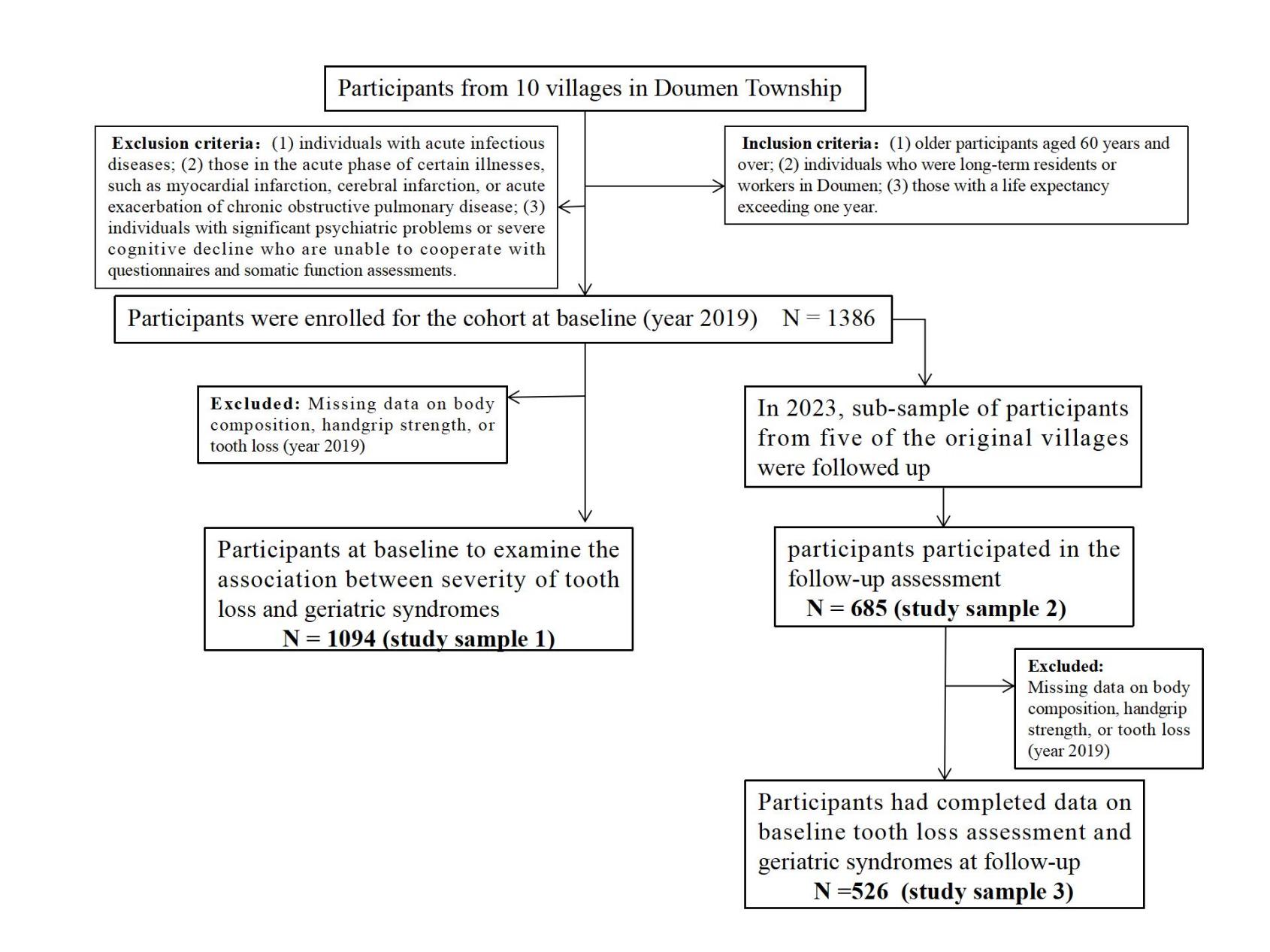
**

**1.Supplementary table S1.** **Characteristics of 529 Participants in the 2019-2023 Cohort in 2023**

| **Characteristic** | N=529 |
| --- | --- |
| Sex, % |  |
| Female | 55.01 |
| Male | 44.99 |
| Age group, % |  |
| 60-70 | 27.98 |
| 70-80 | 53.88 |
| >80 | 18.15 |
| Education level, % |  |
| Illiterate | 54.25 |
| Primary | 38.00 |
| Secondary school or above | 7.75 |
| Sarcopenia, % |  |
| No | 85.63 |
| Yes | 14.37 |
| Having falls over the past year, % |  |
| No | 82.61 |
| Yes | 17.39 |
| Frailty, % |  |
| No | 64.27 |
| Yes | 35.72 |
| Malnutrition risk, % |  |
| No | 66.16 |
| Yes | 33.84 |

1. **Supplementary table S2. Characteristics of the 685 participating followers collected in 2023**

| **Characteristic** | N=685 |
| --- | --- |
| Sex, % |  |
| Female | 55.33 |
| Male | 44.67 |
| Agegroup, % |  |
| 60-70 | 29.34 |
| 70-80 | 52.26 |
| >80 | 18.39 |
| Education level, % |  |
| Illiterate | 52.41 |
| Primary | 36.79 |
| Secondary school or above | 10.80 |
| Smoking, % |  |
| No | 87.45 |
| Yes | 12.55 |
| Drinking, % |  |
| No | 66.57 |
| Yes | 33.43 |
| Sarcopenia, % |  |
| No | 84.23 |
| Yes | 15.77 |
| Number of teeth, % |  |
| ≥21 | 33.72 |
| 11-20 | 24.09 |
| ≤10 | 42.19 |
| Number of chronic disease, % |  |
| None | 22.87 |
| One | 41.02 |
| Two | 24.20 |
| More than two | 11.91 |
| Having falls over the past year, % |  |
| No | 82.34 |
| Yes | 17.66 |
| Frailty, % |  |
| No | 66.42 |
| Yes | 33.58 |
| Malnutrition risk, % |  |
| No | 67.15 |
| Yes | 32.85 |

1. **Supplementary table S3.** **Association between severity of tooth loss and sarcopenia among 1094 participants using cross-sctional data collected at baseline in 2019**

| **Variables** | **Non-adjusted**  **OR (95% CI)** | **Model1**  **OR (95% CI)** | **Model2**  **OR (95% CI)** | **Model3**  **OR (95% CI)** |
| --- | --- | --- | --- | --- |
| Severity of tooth loss and sarcopenia |  |  |  |  |
| No tooth loss | 1 (reference) | 1 (reference) | 1 (reference) | 1 (reference) |
| Having tooth loss that not affecting life | 1.47 (0.76, 2.84) | 1.51 (0.78, 2.92) | 1.47 (0.76, 2.84) | 1.47 (0.76, 2.86) |
| Having tooth loss that affecting life | **2.74 (1.37, 5.48)**** | **2.91 (1.42, 5.95)**** | **2.88 (1.40, 5.89)**** | **2.91 (1.42, 5.97)**** |
| Notes: Model 1 adjusted for age, sex; Model 2 adjusted for age, sex, education; Model 3 adjusted for age, sex, education, drinking, smoking, number of chronic disease.  Data in bold indicate statistically significant values: ***p*<0.001.  Hosmer-Lemeshow goodness-of-fit: all *p* > 0.05. A p-value > 0.05 indicates that the model fits the data well.  CI: confidence interval. | | | | |

1. **Supplementary table S4. Association of initial tooth loss severity with geriatric syndromes in a 4-year follow-up study of 529 participants in 2023**

| **Variables** | **Non-adjusted**  **OR (95% CI)** | **Model1**  **OR (95% CI)** | **Model2**  **OR (95% CI)** | **Model3**  **OR (95% CI)** |
| --- | --- | --- | --- | --- |
| Severity of tooth loss and sarcopenia |  | | | |
| No tooth loss | 1 (reference) | 1 (reference) | 1 (reference) | 1 (reference) |
| Having tooth loss that not affecting life | 0.73 (0.35, 1.54) | 0.67 (0.31, 1.43) | 0.65 (0.31, 1.40) | 0.66 (0.31, 1.41） |
| Having tooth loss that affecting life | 1.77 (0.78, 3.99) | 1.36 (0.59, 3.15) | 1.33 (0.57, 3.18) | 1.27 (0.54, 2.97） |
| Severity of tooth loss and malnutrition risk |  | | | |
| No tooth loss | 1 (reference) | 1 (reference) | 1 (reference) | 1 (reference) |
| Having tooth loss that not affecting life | 1.24 (0.67, 2.28) | 1.21 (0.65, 2.23) | 1.19 (0.64, 2.21) | 1.19 (0.64, 2.21) |
| Having tooth loss that affecting life | **2.88 (1.45, 5.74)**** | **2.81 (1.39, 5.67)**** | **2.80 (1.38, 5.65)**** | **2.68 (1.31, 5.45)**** |
| Severity of tooth loss and frailty |  | | | |
| No tooth loss | 1 (reference) | 1 (reference) | 1 (reference) | 1 (reference) |
| Having tooth loss that not affecting life | 0.77 (0.44, 1.33) | 0.70 (0.40, 1.23) | 0.70 (0.40, 1.23) | 0.68 (0.38, 1.19) |
| Having tooth loss that affecting life | 1.22 (0.64, 2.33) | 0.96 (0.49, 1.88) | 0.96 (0.49, 1.88) | 0.98 (0.50, 1.95) |
| Severity of tooth loss and fall |  | | | |
| No tooth loss | 1 (reference) | 1 (reference) | 1 (reference) | 1 (reference) |
| Having tooth loss that not affecting life | 1.35 (0.61, 2.99) | 1.33 (0.60, 2.96) | 1.34 (0.60, 2.99) | 1.28 (0.57, 2.87) |
| Having tooth loss that affecting life | 1.64 (0.67, 4.01) | 1.42 (0.57, 3.54) | 1.44 (0.58, 3.59) | 1.32 (0.52, 3.36) |
| Severity of tooth loss and total outcome score |  |  |  |  |
| No tooth loss |  |  |  |  |
| Having tooth loss that not affecting life | 1.01 (0.61, 1.66） | 0.91 (0.55, 1.50) | 0.90 (0.54, 1.49) | 0.92 (0.55, 1.54) |
| Having tooth loss that affecting life | **2.25 (1.25, 4.04)*** | **1.82 (1.00, 3.29)*** | 1.81 (1.00, 3.28) | 1.72 (0.94, 3.12） |
| Notes: Model 1 adjusted for age, sex; Model 2 adjusted for age, sex, education; Model 3 adjusted for age, sex, education, drinking, smoking, number of chronic disease.  Data in bold indicate statistically significant values: **p*<0.05, ***p*<0.001.  Hosmer-Lemeshow goodness-of-fit: all *p* > 0.05. A p-value > 0.05 indicates that the model fits the data well.  CI: confidence interval. | | | | |

**2019 Baseline Survey**

**(questions used for the current study)**

**Enrollment Criteria: whether it meets the enrollment criteria □ yes = 1 □ no = 2**

1. Elderly participants at the age of 60 and over.
2. Individuals who were long-term residents or workers in Doumen.
3. Those with a life expectancy exceeding one year.
4. Individuals capable of cooperating in completing the questionnaire

and physical fitness assessments.

**Exclusion Criteria: whether it meets the exclusion criteria □ yes = 1 □ no = 2**

1. Individuals with acute infectious diseases.
2. Those in the acute phase of certain illnesses, such as myocardial infarction, cerebral infarction, or acute exacerbation of chronic obstructive pulmonary disease.
3. Individuals who declined to participate in the study.

**Survey location:** □ province (city) □ County

**Subject No.: □□□□□□□□□□□**

**Date: □□□□/□□/□□**

**Source of Respondents:** 1 = urban community 2 = urban unit 3 = town 4 = rural area 5 = hospital (physical examination center, outpatient service) 6 = other

**Investigator code of this questionnaire: □□□□**

March 2019

**A Basic Information**

**A1 Name:___________**

**A2 Gender:** 1 = male 2 = female

**A3 Age:** ______years old

**A4 Highest education completed:** 1 = illiterate 2 = primary school 3 = junior school 4 = high school 5 = junior college 6 = undergraduate 7 = Master 8 = doctoral

**A5** S**ource of information:** 1 = myself 2 = family 3 = nanny 4 = others

**B Teeth and lifestyle**

**B1 Do you have any missing teeth ?**

1 = No 2 = Yes, but it does not impact daily life 3 = Yes, and it significantly affects daily life.

**B2 Do you drink?**

1 = don't drink alcohol 2 = drink often now 3 = drink often in the past and quit

**B3 Do you smoke?**

1 = never smoke 2 = smoke now (> 5 cigarettes / day) 3 = smoke occasionally 4 = smoke in the past and quit (no smoking for more than 1 month)

**C Chronic disease**

**Do you have the following diseases?**

| Disease | Yes, tick √; no, don't fill in |
| --- | --- |
| hypertension |  |
| diabetes mellitus |  |
| heart disease |  |
| stroke and cerebrovascular diseases |  |
| chronic obstructive pulmonary disease (COPD), |  |
| tuberculosis |  |
| cataract |  |
| chronic nephritis |  |
| cancer |  |
| gastrointestinal diseases |  |
| Parkinson's disease |  |
| falls |  |
| arthritis |  |
| dementia |  |
| metabolic disorder (elevated levels of blood glucose, blood lipid and blood uric acid) |  |
| others, please specify |  |

**2023 Follow-up Survey**

**(questions used for the current study)**

**Survey location:** □ province (city) □ County

**Subject No.: □□□□□□□□□□□**

**Date: □□□□/□□/□□**

**Source of Respondents:** 1 = urban community 2 = urban unit 3 = town 4 = rural area

5 = hospital (physical examination center, outpatient service) 6 = other

**Investigator code of this questionnaire: □□□□**

2023

**A Basic Information**

**A1 Name:___________**

**A2 Gender:** 1 = male 2 = female

**A3 Date of birth:** ____ Year ____ Month ____ Day

**A4 Highest education completed:**

1 = illiterate 2 = primary school 3 = junior school 4 = high school 5 = junior college

6 = undergraduate 7 = Master 8 = doctoral

**A5 Do you smoke?**

1 = never smoke 2 = Have quit smoking 3 = Still smoking

( Please complete if you answered “2” or “3”: Smoking years: ____ years, Average smoking: ____ cigarettes / day )

**A6 Do you drink?**

1 = Never or hardly ever drink 2 = Have quit drinking: ____ years 3 = Drink every day 4 = 3-6 times / week 5 = 1-2 times / week 6 = Less than 1 time / week

**B Oral health**

**B1 How many naturally growing teeth do you still have? (Excluding wisdom teeth, a normal person has 28 teeth)**

1 = < 5 teeth 2 = 5-10 teeth 3 = 11-15 teeth 4 = 16-20 teeth 5 = 21-25 teeth

6 = > 25 teeth

**B2 Do you  have dentures? (Multiple choice possible)**

1 = Without dentures 2 = Dental implants or fixed dentures, ____ teeth 3 = Removable partial dentures 4 = Complete removable dentures.

**B3 Does your oral and dental issue affect your ability to eat?**

1 =  I do not feel that there is much impact or the impact is minimal.

2 = There is still a certain degree of impact.

3 = It severely affects my daily eating, influencing food choices and the ability to chew.

**C Fall history**

**C1 Do you use a cane, walker, or wheelchair for daily walking? (Multiple choices are allowed)**

1 = I can walk independently.

2 = I occasionally use a cane.

3 = I use a cane for an extended period.

4 = I use a walker.

5 = I use a wheelchair.

**C2 Have you experienced a fall in the past year? (A fall is defined as a sudden, involuntary, and unintentional change in body position that results in coming to rest on the ground.) This includes incidents such as slipping, tripping, stumbling, or falling from a level surface.**

1 = Yes 2 = No (If you select no skip the following questions)

**C3 Number of falls in the last year:** ____ time.

**D Chronic disease**

**Do you have the following diseases?**

| Disease | Yes, tick √; no, don't fill in |
| --- | --- |
| hypertension |  |
| diabetes mellitus |  |
| heart disease |  |
| stroke and cerebrovascular diseases |  |
| chronic obstructive pulmonary disease (COPD), |  |
| tuberculosis |  |
| cataract |  |
| chronic nephritis |  |
| cancer |  |
| gastrointestinal diseases |  |
| Parkinson's disease |  |
| falls |  |
| arthritis |  |
| dementia |  |
| metabolic disorder (elevated levels of blood glucose, blood lipid and blood uric acid) |  |
| others, please specify |  |

**FRAIL Scale**


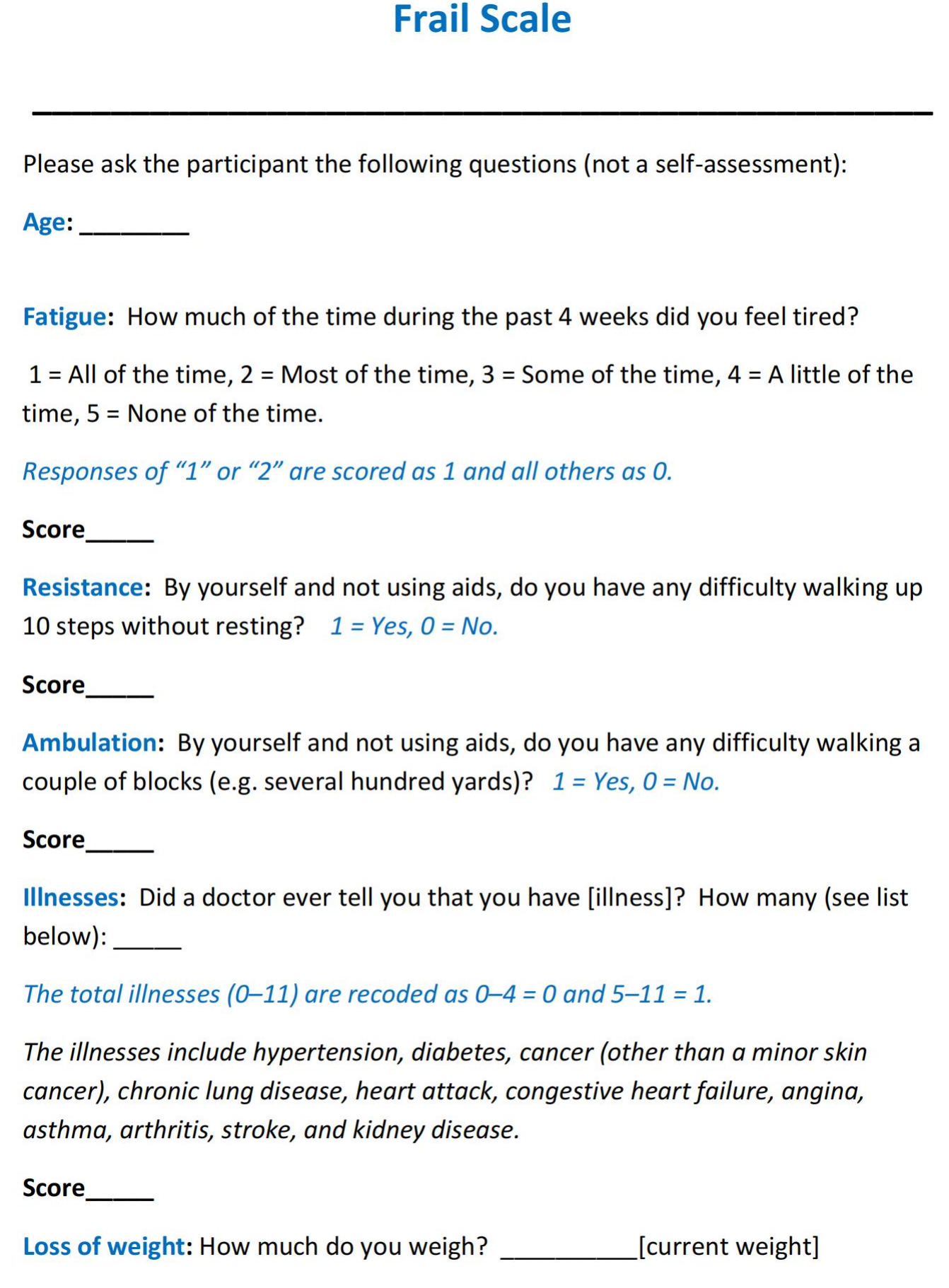


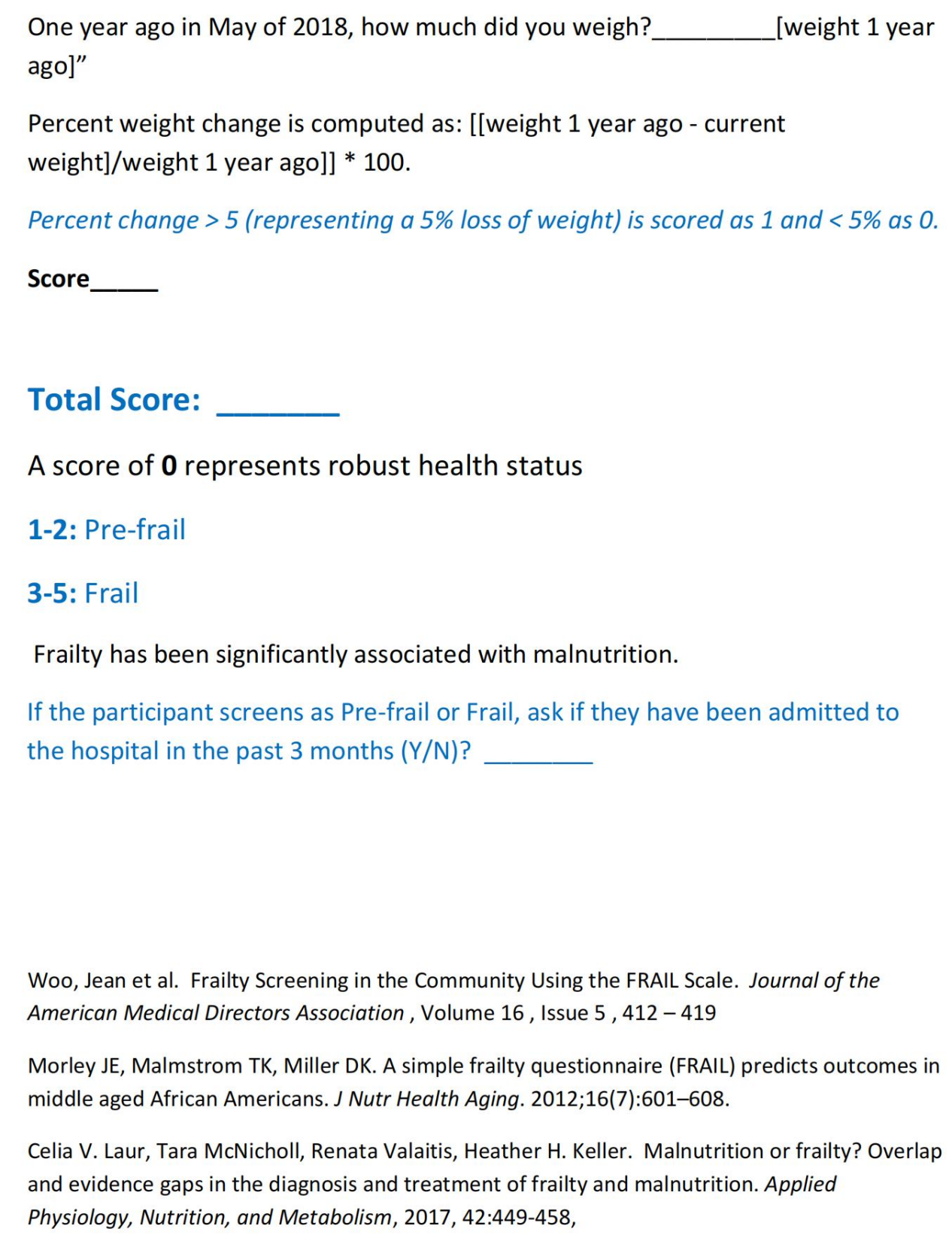


**MNA-SF**


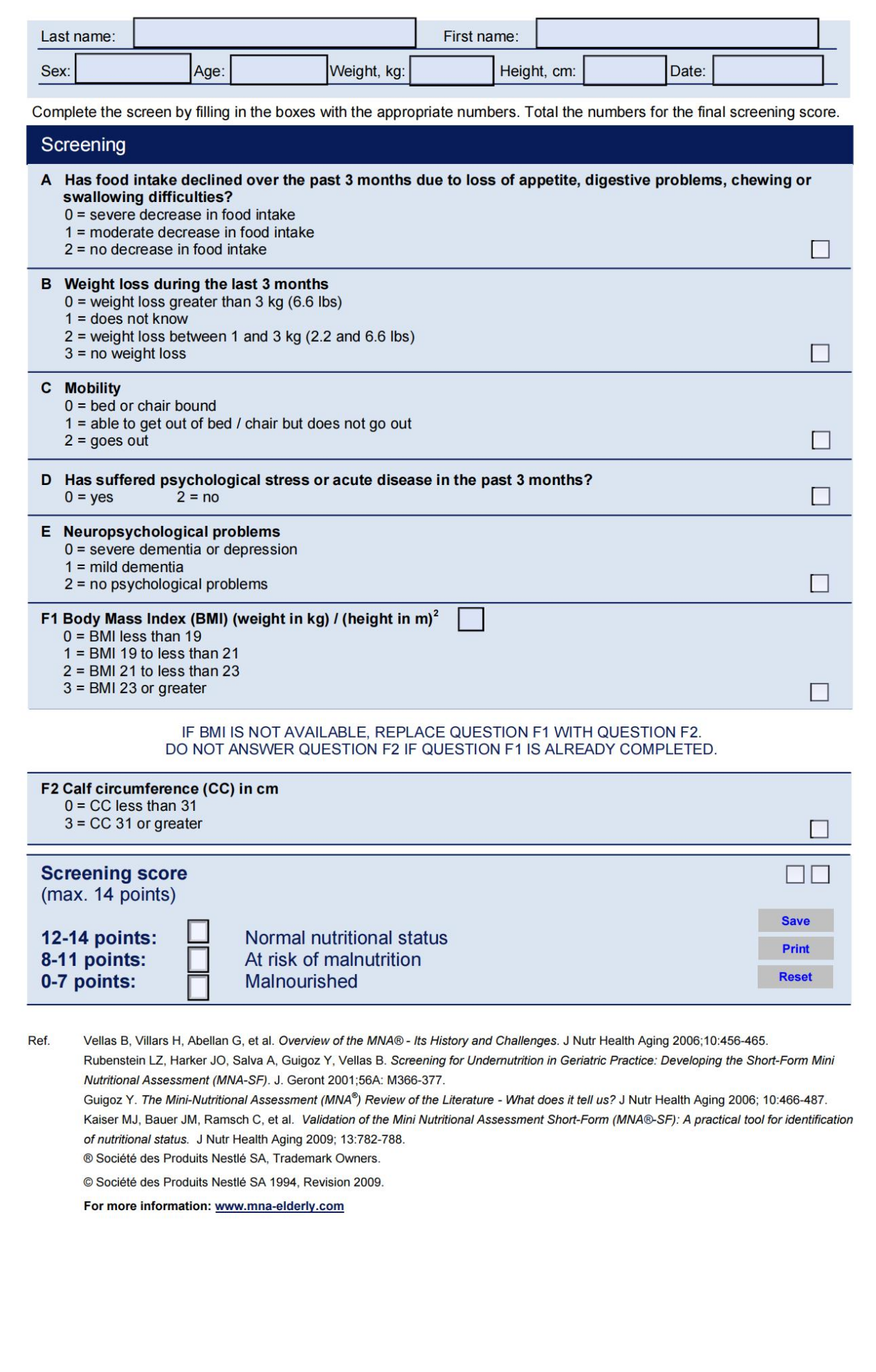

Supplement: Supplementary file 1 — Supplementary Material 1 [file 40520_2025_3032_MOESM1_ESM.docx]
